# Effectiveness and Efficiency of Corral Traps, Drop Nets and Suspended Traps for Capturing Wild Pigs (*Sus scrofa*)

**DOI:** 10.3390/ani11061565

**Published:** 2021-05-27

**Authors:** Joshua A. Gaskamp, Kenneth L. Gee, Tyler A. Campbell, Nova J. Silvy, Stephen L. Webb

**Affiliations:** 1 Noble Research Institute, LLC, Ardmore, OK 73401, USA; jagaskamp@noble.org; 2 Oaks and Prairies Joint Venture, Gene Autry, OK 73401, USA; kennethlgee@gmail.com; 3 East Foundation, San Antonio, TX 78216, USA; tcampbell@eastfoundation.net; 4 Department of Wildlife and Fisheries Sciences, Texas A&M University, College Station, TX 77843, USA; n-silvy@tamu.edu

**Keywords:** capture techniques, feral hogs, human–wildlife conflict, invasive species, population control, smart trap, *Sus scrofa*, trapping, wild boar

## Abstract

**Simple Summary:**

New or revised techniques are being developed to improve management of expanding populations of invasive wild pigs. In southern Oklahoma, we set out to evaluate the success of various trap types: a conventional corral trap design, drop nets developed for capturing other wildlife, and recently developed suspended traps. Suspended traps removed 88.1% of the estimated population of wild pigs, whereas drop nets removed 85.7% and corral traps removed 48.5%. Suspended traps removed one pig for every 0.64 h invested in control, whereas drop nets had a 1.9 h investment per pig and corral traps had a 2.3 h investment per pig. Drop nets and suspended traps removed more of the wild pig population, mainly through whole sounder removal. The suspended trap required the least amount of time per pig removed because of real-time notifications and remote actuation from a cell phone. Drop nets and suspended traps offer greater control and time savings with the use of remote technology, making intensive, large-scale removal of pigs possible. Now, with commercially available technology, corral traps also can be configured to be user operated with real-time monitoring and trapping.

**Abstract:**

Strategic control and eradication programs for wild pigs (*Sus scrofa*) are being developed to help curtail the expanding populations of this invasive, alien species. Drop nets and corral traps have a long history of capturing a multitude of wildlife species, so we evaluated the effectiveness and efficiency of these traps for controlling wild pigs in southern Oklahoma. We also developed and evaluated a suspended metal trap that provided real-time monitoring and deployment to capture animals. Effectiveness of each trap type was estimated as the proportion of pigs removed from the total population, whereas efficiency was calculated based on catch per unit effort (CPUE) (i.e., the number of person hours per pig removal). During 3 years of study (2010–2012), we removed 601 pigs, 296 using drop nets, 60 using corral traps, and 245 using suspended traps. Suspended traps removed 88.1% of the estimated population, whereas drop nets removed 85.7% and corral traps removed 48.5%. CPUE was 0.64 person hours/pig using suspended traps followed by 1.9 person hours/pig for drop nets and 2.3 person hours/pig for corral traps. Drop nets and suspended traps were more effective at removing a large proportion of the population (>85%), mainly through whole sounder removal, but the suspended trap with real-time notifications was the most efficient trap type, requiring fewer person hours to operate.

## 1. Introduction

Invasive wild pig (*Sus scrofa*) populations are expanding rapidly in distribution and abundance across North America [1,2,3], South America [4], Europe [5], and Australia [6]. Expansion has occurred naturally, but this usually occurs over decades [3]. However, wild pig populations also expand through human-mediated translocations [7,8], which then creates localized populations from which natural expansion occurs. When novel, invasive species begin to inhabit a new area, they typically see rapid, exponential population growth because of few regulating factors [9]. For example, with wild pigs, food is often abundant, there are few natural predators, they are generalist, have high survival, and are highly reproductive [5,10]. These features give invasive wild pigs an advantage at becoming well established if control measures are not implemented early during invasion. For this reason, proactive control programs should have well-developed contingency plans that describe strategic and integrative approaches to be most effective at controlling this invasive generalist species.

Since their introduction to the United States and other countries, e.g., [4,5,6], expanding populations of wild pigs have negatively impacted land and natural resources. Their presence has resulted in biodiversity and agricultural losses, depredation of native flora and fauna, destruction of habitat, disease transmission, and other public safety issues [5,6,11,12,13,14,15,16,17,18]. These activities landed the wild pig on the list of the 100 most ecologically destructive invasive species in the world [19]. The wild pig’s reproductive potential [17,20] and adaptability to a broad range of habitats explains persistent populations and continuous damage in the wake of conventional control efforts [21].

Presently, wild pigs are found throughout Oklahoma, USA, with some of the highest densities occurring in the southern part of the state [22]. Historically, wild pigs did not occur in Oklahoma until the late 19th century [23]. Pigs were brought to Texas in the 16th century mainly as a source of food, but later introductions and translocations occurred because of an interest in hunting [23,24]. Wild pigs in Oklahoma likely originated from expanding pig populations in Texas as well as domestic pigs that escaped [23], but are popular for the hunting opportunities they provide.

Along the Red River, in Love County, Oklahoma, wild pigs have caused significant damage to rangelands [25], agricultural areas and specialty crops [26], with a strong threat of disease, especially to livestock and native wildlife [27,28,29]. These issues prompted the need for the development of a control program and trapping systems that would be most effective and efficient at controlling wild pig populations. Control of wild pig populations has been attempted through a broad range of techniques such as hunting, commonly aided by the use of night vision and suppressed firearms, specialty trained bay and catch dogs, snaring, aerial gunning, Judas pigs (i.e., pigs captured, equipped with radio telemetry collars, and then released to track new sounders) and exclusion fencing. Further, many of these techniques are coordinated simultaneously to magnify impact, a strategy known as integrated control [30]. Generally, removal by trapping methods is more effective than other pig control techniques [16].

Some of the most used traps for wild pig removal include box traps and corral traps [31,32,33,34]. These techniques use bait to attract pigs to an area where an enclosure is set up and pigs are trapped by activating a gate or trigger. However, there are many issues that may limit the efficacy of traditional trapping systems, such as trap shyness, non-target captures, false triggers, failure to capture whole sounders, and subsequent education of pigs in close proximity. There is a long history of using traditional traps, such as corral traps, for capturing wild pigs because they are readily available commercially and are familiar to wild pig trappers. Therefore, we used this trap design to evaluate alongside two newer trap types for wild pig control (i.e., drop nets and suspended traps).

Drop nets have a long history of capturing species such as white-tailed deer (*Odocoileus virginianus*) and wild turkey (*Meleagris gallopavo*) [35,36]. Casual experience using drop nets to capture wild pigs indicated that drop nets might be a viable alternative to conventional trapping techniques [37], but see [18]. One of the major advantages observed early on was that whole sounders of wild pigs could be captured under one net, and animals did not appear to exhibit the same signs of trap shyness as they did to more traditional traps. During evaluation of drop nets for wild pig control, we developed a suspended trap that was portable, rugged (metal), and allowed real-time communication where the trap was activated by the trapper. The suspended trap was evaluated after completion of experiment 1 (see below) that evaluated drop nets and corral traps.

Herein, we evaluated three trap types (i.e., corral traps, drop nets, and suspended traps) for their effectiveness at large-scale control of wild pigs. Effectiveness was measured as the proportion of the population removed based on population size estimates. We also estimated trap efficiency among the three trap types where efficiency is the total amount of person hours required to capture one pig, considering all activities required to construct, monitor, and trap wild pigs. We used catch per unit effort for calculating efficiency. The total cost of each trap type was also reported.

## 2. Materials and Methods

### 2.1. Study Area—Experiment 1

**Figure 1:**
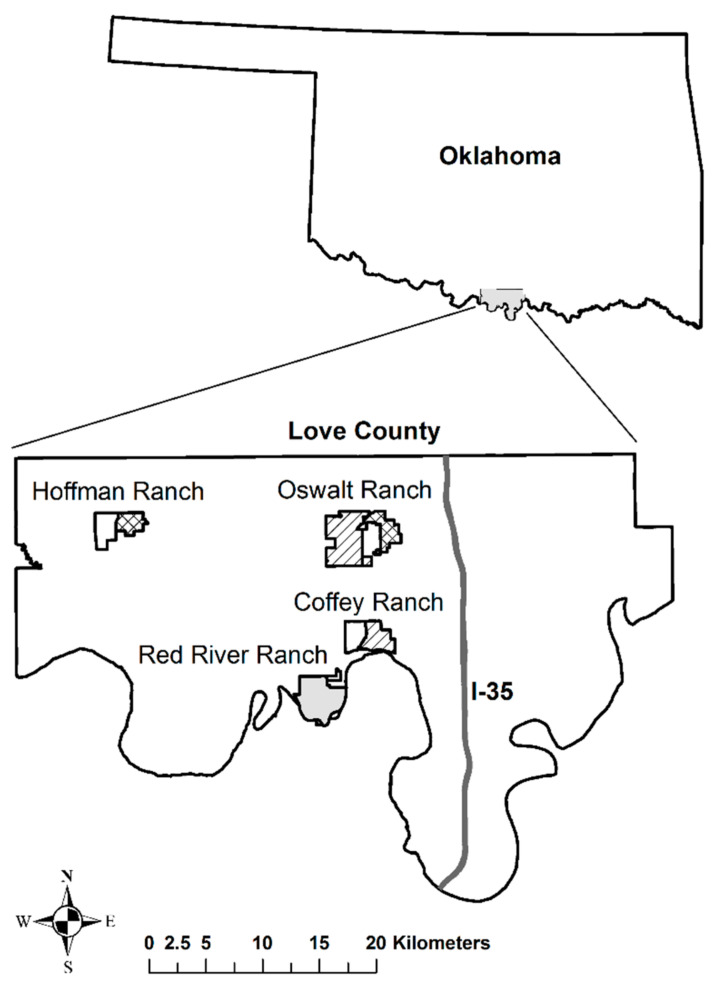
Study area map depicting the Noble Research Institute Oswalt Road Ranch (ORR; 2027.9 ha), Noble Research Institute Coffey Ranch (CR; 1010.6 ha), Noble Research Institute Red River Ranch (RRR; 1316 ha; experiment 2 [solid gray polygon]), and the Hoffmann Ranch (HR; 945.2 ha) located in Love County, Oklahoma. Experiment 1 study sites (ORR, CR and HR) were divided into two units, resulting in six total units where control (i.e., no harvest [open, hollow polygon]) and trapping (i.e., corral traps [crosshatch pattern] and drop nets [dashed lines]) were assigned at random; two units were assigned to each respective treatment and control. Corral traps (east unit; 592.2 ha) and drop nets (west unit; 1435.7 ha) were installed on ORR, drop nets (east unit; 579.7 ha) and a no harvest control (west unit; 431.5 ha) at CR, and corral traps (east unit; 415.8 ha) and a no harvest control (west unit; 529.4 ha) at HR. During Experiment 2, suspended traps were deployed across ORR, CR and RRR in 2012 to determine efficacy and effectiveness.

Experiment 1 assessed efficiency and effectiveness of drop nets and corral traps across three sites: Noble Research Institute Oswalt Road Ranch (ORR; 2027.9 ha), Noble Research Institute Coffey Ranch (CR; 1010.6 ha), and the Hoffmann Ranch (HR; 945.2 ha) in Love County, Oklahoma (Figure 1). The study sites are in the Cross Timbers and Prairies eco-region, which is characterized by a mixture of wooded areas and openings [38]. See [39] for a complete description of vegetation communities across the three study sites.

Study sites were divided into two units (Figure 1), resulting in six total units where control (i.e., no trapping) and trapping (i.e., corral trap and drop net) were assigned to the units at random. Corral traps (east unit; 592.2 ha) and drop nets (west unit; 1435.7 ha) were installed on ORR, drop nets (east unit; 579.7 ha) and a no trapping control (west unit; 431.5 ha) at CR, and corral traps (east unit; 415.8 ha) and a no trapping control (west unit; 529.4 ha) at HR (Figure 1; also see Table 1 in [25]). The placement of trap type in each unit remained unchanged during the second year of trapping and removal.

### 2.2. Study Area—Experiment 2

Experiment 2, assessing the efficiency and effectiveness of a suspended trap, was conducted on CR, ORR and the Noble Research Institute’s Red River Ranch in southern Love County, Oklahoma (Figure 1). For experiment 2, ranches were not split into multiple units, but were trapped as contiguous parcels. RRR is a 1385.2-ha demonstration and research ranch on the northern edge of the Red River, and occurs in the Cross Timbers and Prairies eco-region. Compared to the other sites consisting of primarily native rangeland, RRR is also characterized by introduced pasture (Bermuda grass; *Cynodon dactylon*) to support cattle grazing, and native and improved pecan operations [26].

### 2.3. Camera Surveys

Prior to trapping for experiments 1 and 2, camera surveys were conducted annually from January to February at each study site for the presence of wild pigs. Still and video cameras (Cuddeback NoFlash, Non Typical, Inc., Green Bay, WI, USA) were used to identify potential trapping locations, estimate population demographics, and individually identify unique pigs (see 2.5. Effectiveness). Cameras were randomly allocated across ORR (*n* = 40; 1 camera/52.3 ha), CR (*n* = 26; 1 camera/39.4 ha), HR (*n* = 23; 1 camera/40.4 ha) [18], and RRR (*n* = 20; 1 camera/69.3 ha). Camera sites were baited daily with whole kernel corn for 14 days; the first seven days allowed wild pigs to find and acclimate to the bait site, and days 8 to 14 were used to conduct a formal camera survey. After completion of camera surveys, all sites were abandoned and not baited to allow pigs to redistribute before trapping. Trap sites were then selected based on wild pig presence that was documented during the camera survey. These trap sites were baited for seven days with whole kernel corn, and if any pigs visited the baited trap site for ≥3 consecutive days then a trap was installed.

### 2.4. Trapping

**Figure 2:**
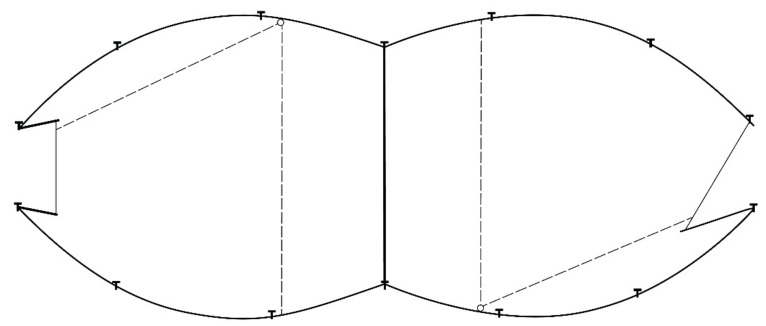
Corral trap configuration (9.75 m (l) × 2.4 m (w) × 1.5 m (h)) that includes saloon-style gate (**left**), single spring gate (**right**), and trip wires (dashed lines) used on Oswalt Road and Hoffmann ranches, Love County, Oklahoma, USA to trap wild pigs (*Sus scrofa*).

**Figure 3:**
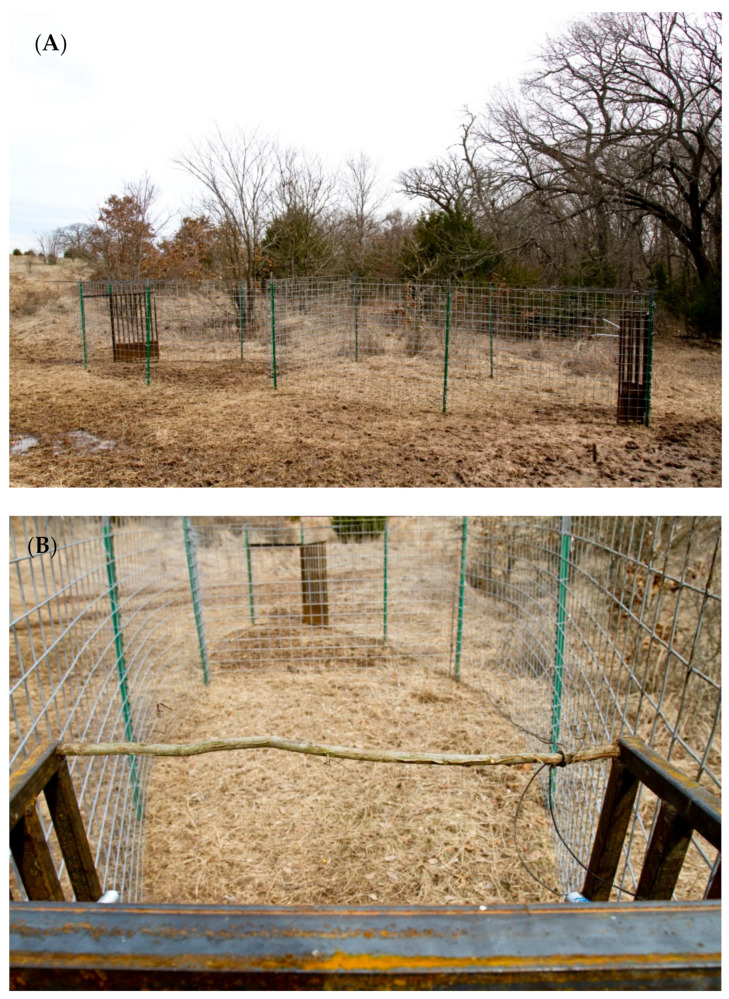
Corral trap (9.75 m (l) × 2.4 m (w) × 1.5 m (h)) showing overall design, adjoining compartments, and two different gate openings (single spring gate on left and saloon-style gate on right) (**A**). Interior view of the adjoining compartments with the saloon-style gate in the foreground, and the single spring gate in the background (**B**). Photographs courtesy of Noble Research Institute, LLC (Ardmore, OK, USA).

Corral traps [10] were 9.75 m (l) × 2.4 m (w) × 1.5 m (h) and constructed of metal t-posts and cattle panels (10 × 10 cm mesh size) (Figure 2). Traps also consisted of two adjoining compartments with two different gate openings (i.e., single spring and saloon-style gates) facing opposite one another (Figure 3). Corral trap gates were animal activated via trip wire. These traps were capable of capturing additional pigs in the adjacent compartment once one half was already tripped; the traps also were capable of capturing additional pigs through previously activated gates.

**Figure 4:**
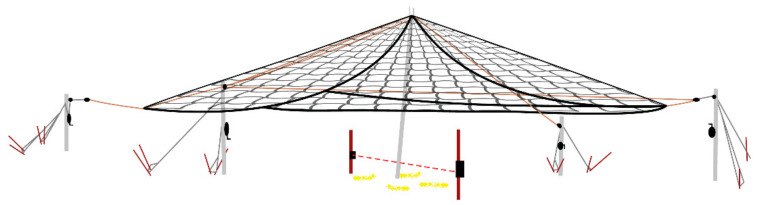
Drop net configuration including net (18.3 m (l) × 18.3 m (w)), center pole (6.1 m (h)), rope harnesses, support poles, deadmen anchors, infrared trail monitor, and bait placement used on Coffey and Oswalt Road ranches, Love County, OK, USA to trap wild pigs (*Sus scrofa*).

**Figure 5:**
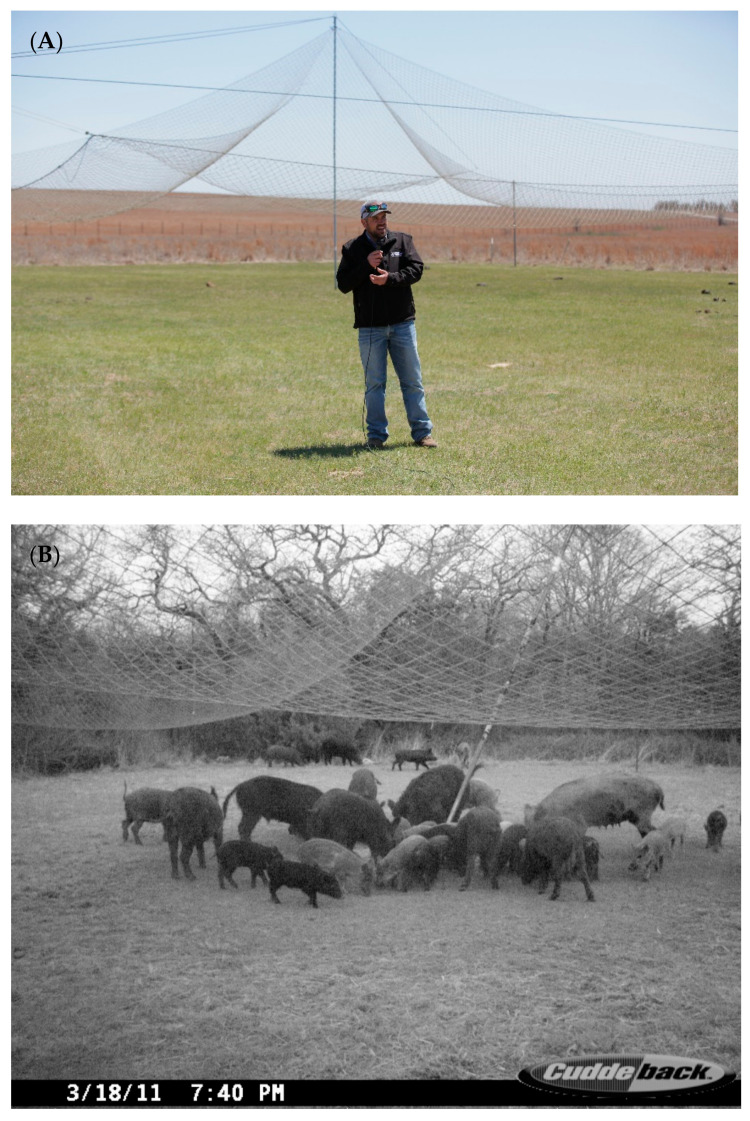
Wide angle view of the drop net used to capture wild pigs (*Sus scrofa*) in Love County, OK, USA (**A**). Sounder of wild pigs consuming corn under a drop net <30 min after the net was erected and baited; photograph captured with Cuddeback digital camera (Cuddeback, De Pere, WI, USA) (**B**). Photographs courtesy of Noble Research Institute, LLC (Ardmore, OK, USA).

Drop nets [36] were modified based on designs by [35], which required human presence to operate. The system incorporates a net (18.3 m (l) × 18.3 m (w)), center pole (6.1 m (h)) multiple rope harnesses, a release mechanism, solenoids, batteries, and a line-of-sight remote control for triggering the net to drop (Figure 4 and Figure 5). Trailmaster active infrared trail monitors (TM 1050, Goodson & Associates, Inc., Lenexa, KS, USA), in combination with a radio frequency transmitter and 2-way radio, were used to monitor activity under nets thereby eliminating the need for constant observation. The drop net system was also equipped with a remote-controlled infrared-filtered spotlight (Trailmaster, Goodson & Associates, Inc., Lenexa, KS, USA) to facilitate nocturnal use.

**Figure 6:**
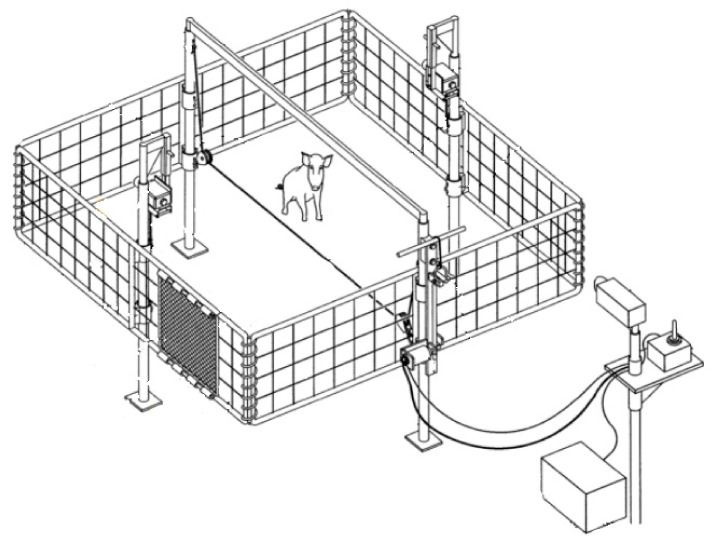
Suspended trap configuration (4.9 m (l) × 4.9 m (w) × 1.5 m (h)) used on Coffey, Oswalt Road and Red River ranches, Love County, OK, USA to trap wild pigs (*Sus scrofa*). Diagram courtesy of Noble Research Institute, LLC (Ardmore, OK, USA).

**Figure 7:**
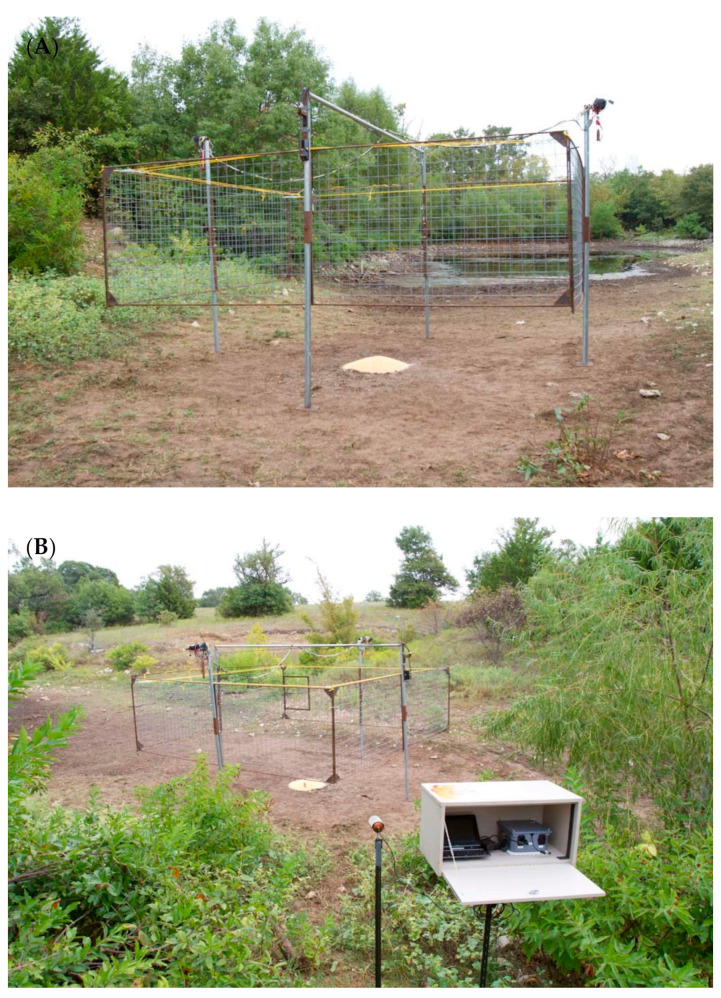
Suspended trap used to capture wild pigs (*Sus scrofa*) on Coffey, Oswalt Road and Red River ranches, Love County, OK, USA (**A**). Electronics, camera and hardware (in foreground) used to provide images of animals under the trap to the user via internet using cellular data (**B**). The trapper also used this system of hardware and software to send a signal for the trap to drop. Photographs courtesy of Noble Research Institute, LLC (Ardmore, OK, USA).

Suspended traps (Figure 6 and Figure 7) measured 4.9 m (l) × 4.9 m (w) square, and like corral traps, they were 1.5 m tall when sitting on the ground. The 4 sides were framed with steel rod and pinned together to form a rigid square cage; each of the four sides consisted of 10 × 10 cm mesh size cattle panels. The suspended trap cage could be elevated using a pair of rope harnesses and pulleys acting against a pole mast in the center of each side. The trap was hoisted approximately 1 m using two (800 lb) 12 volt winches (Warn Industries, Clackamas, OR, USA) attached directly to a truck battery. This system also used similar solenoids and batteries described in the drop net system [28,32], but were connected to a portable laptop computer with a USB relay that activated the trap. The computer was internet capable, which used cellular 3G communication for connection, allowing the user to monitor an attached webcam in real time. An ad hoc software application was configured on the computer to relay notifications of animal activity to the user via cellular communication when motion was detected on the webcam. The user could communicate with 1 of the USB relays to flood the trap area with infrared light, while the other USB relay was used to activate the solenoids used to drop the suspended trap cage. The infrared lights and user activated relay to the drop solenoids gave the user discretion over when to drop the trap to capture wild pigs.

After corral traps were set and baited, the gates were tied open for ≥3 days to allow pigs to become familiar with the trap; during this time, bait was replenished daily. During the non-operational period (first three days), drop nets were baited daily with corn starting at the center pole and extending in lines to the edge of the net; drop nets were baited around the center pole only during trapping. During the non-operational period, suspended traps were baited daily with corn in the center of the trap with lines of corn extending to each side. Suspended traps were baited only in the center during trapping. After three days, trapping was initiated and traps were baited daily, pigs removed upon capture, and traps reset until cameras showed no further pig activity for ≥5 consecutive days. Wild pig harvest or removal by other means was not allowed on the study sites during the study period. Wild pigs were euthanized upon capture via a shot to the brain from a 5.6 mm (0.22 inch) calibre rifle. Animal capture and euthanasia procedures were conducted in accordance with Animal Use Protocol 2008-160 issued by Texas A&M University.

### 2.5. Effectiveness

Following other studies that used remote game cameras to census wildlife based on identifiable subjects (e.g., white-tailed deer) [40,41], we developed a method for estimating the identifiable segment of wild pig populations to test trap effectiveness. Effectiveness refers to the proportion of the population removed based on population size as estimated by the Lincoln–Petersen method. The initial “capture” was based on pigs being captured on camera, and the “mark” occurred for pigs with uniquely identifiable features such as color, pelt patterns, scars, deformities or any combination thereof. A “recapture” occurred when a pig was captured and removed. Using the Lincoln–Petersen method [42,43,44], we estimated population size as:

P = N × (M/R)(1)

where P is population size, N is total number of new individuals captured during trapping, M is the initial number of marked individual pigs, and R is the number of individually marked pigs recaptured during trapping. Based on study design and the species, assumptions were met in that (1) all individuals are equally catchable because corn was provided as an attractant, (2) individuals do not lose marks because we used natural markings, and (3) marking and capture were performed over a relatively short period (from late January to April) so the population could be consider closed. Trap effectiveness (i.e., the ratio of N/P) was calculated by study site (i.e., ranch) and trap type (i.e., drop net [*n* = 15] and corral [*n* = 11]) for each year (i.e., 2010–2012).

### 2.6. Efficiency

We calculated efficiency for each trap type: corral trap, drop net and suspended trap. We recorded time associated with each activity (i.e., baiting, trap construction, trapping, and trap disassembly) for each trap type and individual trap. Time records started when the vehicle entered a ranch and ended when it left the ranch. Data collected included date, ranch, treatment (trap type), trap site, activity (e.g., baiting, trap construction, trapping, and trap disassembly), number of people present, and total time on each activity. For each capture of pigs, we recorded total number of pigs, number of males and females, and body mass; body mass was used to classify pigs as piglets or juveniles (<27 kg) and subadults or adults (≥27 kg) [12]. These records were used to calculate catch per unit effort (CPUE) for each trap type and individual trap. For individual trap site, CPUE was calculated as the total number of pigs captured at an individual trap site divided by the total time invested in control at each respective trap site. For trap type, CPUE was calculated as the total number of pigs captured across all trap sites for a respective trap type divided by the total time invested in control of pigs via each trap type.

## 3. Results

### 3.1. Trapping

During experiment 1, we captured 222 wild pigs in 2010 and 134 in 2011 (N = 356). We caught 173 and 123 pigs using drop nets in 2010 and 2011, respectively (*n* = 296). In 2010, we caught 49 pigs in corral traps, but only 11 pigs in 2011 (*n* = 60). Mean number of captured pigs was 9.6 (±9.0 SD) and 3.9 (±3.7 SD) in drop nets and corral traps, respectively. The largest single capture event in a drop net was 27 individuals, whereas the largest single capture event in corral traps was 14 individuals. Juveniles (104 females; 93 males) accounted for 55% of the pigs captured (*n* = 197); of the 159 adults, 97 were females, 60 were males, and two did not have sex assigned. Escapes were observed in both trapping systems. Twenty-nine pigs escaped from drop nets and 11 escaped from corral traps. We used two types of trap doors for the corral trap (saloon and single spring gates); 32 pigs were captured using saloon gates and 28 pigs were caught using single spring gates. Although both gate types allowed further capture of pigs once the gate was closed, we did not document any pigs being captured in this way.

During experiment 2, we captured 245 pigs in 2012 across CR, ORR and RRR using suspended traps. Of the 245 wild pigs caught, eight pigs escaped before euthanasia, so weight could not be recorded for seven pigs and sex for six of the pigs. The mean number of pigs captured in 30 unique trapping events using the suspended trap was 8.2 (±7.9 SD). Juvenile captures (*n* = 127) made up just over half (53.4%) of captures compared to adults (*n* = 111; 46.6%). Of the 237 pigs where sex and age class could be assigned, there were 64 juvenile females, 64 adult females, 63 juvenile males, and 46 adult males. The largest capture was 30 pigs using a suspended trap.

### 3.2. Effectiveness

Effectiveness by trap type was 85.7, 48.5, and 88.1% for drop nets, corral traps, and suspended traps, respectively (Table 1). We captured 90.0 and 81.3% of the identifiable pig population using drop nets in 2010 and 2011, respectively (Table 1). With corral traps, we captured 60.6% of the identifiable pig population in 2010 and 36.4% in 2011 (Table 1). Suspended traps were only evaluated in 2012, so effectiveness for each ranch was 80.7% for CR, 95.1% for ORR, and 85.0% for RRR (Table 1).

### 3.3. Efficiency

During the 3 years of study, and across 35 total trap sites (*n* = 15 drop net sites, *n* = 11 corral trap sites, *n* = 9 suspended trap sites), we recorded 2056 activity records for baiting, trap construction and disassembly, trap observation, and wild pig euthanasia and disposal. It took 2.6 person hours to set up corral traps, 1.66 person hours for drop nets, and 0.76 person hours for suspended traps. Driving t-posts was the most time consuming portion of drop net set up, which at times required drilling holes for t-post insertion. Wiring panels to t-posts was the most time consuming activity when building corral traps. Computer and electronic integration and testing required the most time when setting up suspended traps.

Trap observation was required for drop nets and suspended traps; a human trapper was required to be physically present at drop nets but not for suspended traps. Drop net observation required an average of 3.9 person hours, or 49% of total time, regardless of a successful capture (Table 2). Trap observation time was not recorded for suspended traps because a human trapper was not required to be physically present at the trap site (Table 2). A real-time alert was sent to the trapper’s phone, allowing the trapper to see what was under the trap. Then, the trapper could send a signal to drop the trap after confirming wild pig presence.

Baiting also constituted a large proportion of total time for each trap type: 30% for drop nets, 58% for corral traps, and 66% for suspended traps (Table 2). However, trap observation time was not calculated for suspended traps, albeit the time in this activity was minimal for suspended traps. CPUE was 2.4 person hours/pig in 2010 using drop nets, which dropped to 1.2 person hours/pig in 2011. CPUE using corral traps was similar to drop nets in 2010 (2.4 person hours/pig), and was 1.7 person hours/pig in 2011. Across years, CPUE was lower (i.e., more efficient) using suspended traps (0.64 person hours/pig) followed by drop nets (1.9 person hours/pig) and then corral traps (2.3 person hours/pig).

## 4. Discussion

Corral traps have a long history of capturing wild pigs, whereas drop nets have been used to capture a wide range of species [18] but see [36]. This study also evaluated a relatively new trap design for capturing wild pigs, a suspended metal trap. Drop nets and suspended traps have not been critically evaluated for their effectiveness, efficiency and application for removing wild pigs. Suspended traps and drop nets were effective at removing 88.1% and 85.7% of the total estimated pig population, respectively. Suspended traps took the least time to set up (0.76 person hours), and were most efficient at capturing pigs, requiring 0.64 person hours/pig captured. Drop net efficiency was 1.9 person hours/pig, taking 1.66 person hours to set up, whereas corral traps took 2.6 person hours for set up, with an efficiency of 2.3 person hours/pig captured. All trap types can be moved to new locations, but based on set up time, suspended traps may offer a unique advantage when capturing wild pigs across large areas. When traps require less set up time, trappers can invest in fewer systems to reduce long-term costs of removal, resulting in a higher return on investment. Efficiency and effectiveness are enhanced with user-activated trap systems (i.e., drop nets and suspended traps), but even more so when coupled with real-time, remote monitoring and triggering such as was the case for suspended traps. These two designs allowed trappers to be more selective, with the ability to capture whole sounders (Figure 4), resulting in greater population reduction. However, most currently available trap designs, including corral traps, can be retrofitted with commercially available technology, such as cellular communication, remote monitoring and trigger systems, to make trapping more efficient and effective. 

To estimate effectiveness (i.e., the proportion of the population removed based on population size), we had to estimate population size. Previous studies have used remotely triggered cameras to uniquely identify (“mark”) individual subjects from antlers, stripes, coat patterns and more [40,41,45,46,47,48]. Wild pigs vary considerably in their coat patterns and color [10], so we “marked” individually identifiable pigs during camera surveys based on coat patterns and colors, and other physically unique traits (e.g., scars, cuts, malformations). Although time consuming, we found that individual pigs varied enough in physical traits that we could track their presence or absence at individual trap sites, and confidently assign unique individuals as “recaptures” during removal. This allowed us to estimate population size using a traditional capture–recapture method. Additionally, having information on unique individuals allowed us to estimate the percentage of the marked population that was removed. Similar to other remote camera surveys that rely on uniquely marked individuals to estimate population demographics, camera surveys of wild pigs to estimate population size using capture–recapture methods is feasible.

Wild pigs are highly fecund because they reach sexual maturity at a young age, have multiple, large litters per year, and have high survival rates [5,10]. For these reasons, populations can expand rapidly, potentially doubling every five years [20,49]. To prevent further growth, and to maintain a stable population size, as much as 66% of the wild pig population may need to be removed annually [49]. However, another report estimates that it may require removal of 80% of the population to slow population growth [50]. Intensive removal of wild pigs can lead to reduction in damage [25,51] even if the targeted level of population control is not accomplished. In these instances, it may not be cost effective to try to reach a high target level of removal, but rather consider reductions in damage costs as part of total cost effectiveness [51]. However, if the goal is population maintenance or reduction, then population control with drop nets and suspended traps reached the critical thresholds of removal (at either 66% [49] or 80% [50]). In fact, effectiveness using these two trap types was 6–8% greater than the highest level of control reported for population reduction.

There are several reasons why drop nets and suspended traps can remove large portions of the wild pig population. First, these trap designs were able to catch whole sounders, cf. [18]; a primary factor influencing effectiveness and efficiency. On many instances, we captured entire sounders using these two trap systems, some as large as 30 individuals. Although pigs escaped all trap types, average capture size of sounders was 2.5- and 2.1-fold larger with drop nets and suspended traps than corral traps, respectively. Although corral traps had gates that allowed additional pigs to enter the trap after it had been activated (Figure 2 and Figure 3), we did not observe any additional captures in this way, which reduces the ability to capture a whole sounder. 

Wild pigs are wary [10], so we expect some individuals to be more trap shy than others [31]. A second benefit of drop nets and suspended traps is they are less intrusive visually to animals. Most of the infrastructure of these trap types is well above ground level. Drop net only had posts (~5.1 cm diameter) at each of the four corners of the net and one at the center of the net around which corn was baited (Figure 4 and Figure 5). The suspended trap (Figure 6 and Figure 7) only had posts (~5.1 cm diameter) at the center of each of the four sides. Therefore, there were not any confined entry or exit points, trap thresholds to cross, or paneling or fencing at ground level for these trap designs. In comparison, wild pigs may not enter corral traps because they perceive confinement of the trap, have obstructed views, hear greater noise of pigs near or entering the trap, or remember a previous interaction with the same or similar trap. The observed noise and panic associated with trapping events in corral traps may cause pigs that are outside of a trap when the gate is tripped to be frightened, resulting in trap shyness from any negative experience. On the other hand, wild pigs did not seem to exhibit the same timid behavior at drop nets or suspended traps. This again could be due to how traps are constructed and the fact that pigs are not noticing or associating an overhead net or trapping device with as much danger as a traditional trap. We observed that wild pigs regularly walked under drop nets and suspended traps within the first 24 h after the trap was set into place. However, we do note that the area receiving the drop net or suspended trap was baited for ≥7 days before set up; the same pre-baiting process was performed for corral traps but pigs still showed hesitation to entering.

Lastly, drop nets and suspended traps were more effective because they were user activated, cf. [18]. The corral traps used in this study, on the other hand, are animal activated, so there is no control over when a trap is triggered. For example, with most animal-activated traps, there is a higher probability of non-target captures, especially when using certain baits like corn [52,53]. False triggers and the capture of only a portion of the larger group also are factors limiting efficacy of animal-activated traps. However, adding commercially available technology to corral traps, allowing the traps to be user activated, could aid in the capture of whole sounders and a reduction in false triggers. Although drop nets required personnel to monitor the traps, what was lost in personnel time was made up for with greater selectivity over when a net was dropped. User-activated drop nets eliminated guesswork and effectively increased number of animals per capture. The suspended trap also had another advantage over drop nets, even though both trap systems are user activated. The suspended trap was designed with real-time communication to send notifications that animals were under the trap. The user only had to wait for a text notification to view the picture, and then utilize real-time video and cellular data transmission to optimize the trap drop. This real-time system greatly reduced total person hours required for trapping with this method. However, technological solutions are not always user friendly, so users would need to invest time in learning how to configure and troubleshoot any computer or electronic issues.

Although we have reported on the effectiveness and efficiency of three common trap types, users also may want to use methods developed herein to evaluate other trapping or control methods. We used catch per unit effort (CPUE), the number of pigs captured for each trap type divided by time spent on each trapping method, as a measure of efficiency. Using game cameras and uniquely identifiable individuals, we estimated population size using the Lincoln–Peterson estimator, and then estimated effectiveness as the proportion of the population removed based on total estimated population size. For instance, effectiveness of aerial gunning has not been quantified rigorously in areas with similar vegetation and topography that we encountered, but in certain areas, and under varying densities of wild pigs, aerial gunning may be more efficient or effective. Aerial gunning from a helicopter removed 49.3 wild pigs per hour at an initial density of 1.78 pigs/km^2^, but only 8.8 pigs per hour in an area with a lower density (0.62 pigs/km^2^) [50]. However, the choice of control method will be dependent on area available for trapping, habitat and topographic features, personnel availability, and financial resources. For use in more rugged and forested landscapes, similar to those in our study, we found the three trap systems fit the circumstances, but that suspended traps were the most effective and efficient.

Financial costs will be a major factor in the trap design that users choose, although cost alone should not dictate choice of trapping system. The cost of the 3 trap systems, priced around the time of this study in 2011, was approximately $3500 for a drop net, $500 for a corral trap, and $1500 for a suspended trap including all technology. The suspended trap also had the added expense of cellular data, costing $50/month.

## 5. Conclusions

Most conventional trapping methods, including corral traps, are not effective at controlling wild pigs at the scale necessary to have significant, long-term effects on reducing populations [32,54]. However, drop nets and suspended traps may provide greater control over a larger area than corral traps, thus reducing the number of traps necessary per landowner or association of landowners. Drop nets and suspended traps caught more pigs in less time, allowing more frequent relocation to prioritize areas receiving damage, or to have a greater impact on distributed pig populations. However, other practices also contribute to greater effectiveness and efficiency. We recommend baiting for ≥7 days before setting traps to reduce trap wariness, even though pigs may be more wary of corral traps than drop nets or suspended traps in general. With the development of technology, like that used for the suspended trap, or with other technologies such as cellular game cameras, landowners may be able to monitor trap systems in real time to reduce the amount of effort monitoring traps. Similar to the suspended trap used herein, other technology on the commercial market also can make trap systems user activated from remote locations, further reducing effort but at a greater initial cost.

## 6. Patents

Gaskamp, J.A.; Gee, K.L.; Rhines, S.P. (2016) Systems and methods for trapping animals. United States Patent, US Patent 9,237,743, filed 18 April 2014 and issued 19 January 2016.

Gaskamp, J.A.; Gee, K.L.; Rhines, S.P. (2017) Systems and methods for trapping animals. United States Patent, US Patent 9,668,467, filed 29 October 2015 and issued 6 June 2017.

Gaskamp, J.A.; Gee, K.L. (2018) Systems and methods for trapping animals. United States Patent, US Patent 10,076,109 filed 14 February 2012 and issued 18 September 2018.

Gaskamp, J.A.; Gee, K.L. (2019) Systems and methods for trapping animals. United States Patent, US Patent 10,470,454, filed 23 March 2018 and issued 12 November 2019.

## Figures and Tables

**Table 1:** Trap effectiveness for capturing wild pigs (*Sus scrofa*) by ranch for each trap type (drop net, corral trap and suspended trap) and year (2010–2012) on Coffey, Hoffman, Oswalt Road, and Red River ranches, Love County, Oklahoma, USA.

Year	Trap	Ranch	Marked (M; *n*)	Captured (N; *n*)	Recaptured (R; *n*)	Population (P; *n*)	Effectiveness (N/P)
2010	Drop net	Coffey	55	55	51	59.3	0.927
		Oswalt Road	63	55	55	63	0.873
	Corral	Hoffmann	11	8	8	11	0.727
		Oswalt Road	17	15	8	31	0.484
2011	Drop net	Coffey	51	50	47	54.25	0.922
		Oswalt Road	54	39	38	55.4	0.704
	Corral	Hoffmann	11	4	4	11	0.364
		Oswalt Road	0	0	0	0	--
		Coffey	34	26	26	34	0.807
2012	Suspended trap	Oswalt Road	42	40	40	42	0.951
		Red River	21	40	18	46.5	0.85

‘Marked’ (M) refers to the number (*n*) of uniquely identifiable wild pigs from photographs using the respective trap on each ranch during each year (2010–2012). ‘Captured’ (N) refers to the total number (*n*) of wild pigs caught irrespective of whether they were uniquely marked. ‘Recaptured’ (R) refers to the number (*n*) of uniquely identifiable pigs that were caught and removed from the total number of marked pigs. ‘Population’ size was estimated using the Lincoln–Petersen estimator (P = N × (M/R)). ‘Effectiveness’ is calculated as Captured/Population (N/P) and is an estimate of the proportion of the wild pig population removed for the respective trap type on each ranch during each year.

**Table 2:** Percentage (%) of time spent on various activities while trapping wild pigs (*Sus scrofa*) using drop nets (Coffey and Oswalt Road ranches; 2010–2011), corral traps (Hoffmann and Oswalt Road ranches; 2010–2011), and suspended traps (Coffey, Oswalt and Red River ranches; 2012) in Love County, Oklahoma, USA. Total time for each method was 9.79, 7.2 and 2.92 h for drop nets, corral traps and suspended traps, respectively.

Activity	Drop Net (%)	Corral Trap (%)	Suspended Trap (%)
Construction/maintenance ^1^	17	36	26
Baiting ^2^	30	58	66
Trap observation ^3^	49	0	^5^
Removal ^4^	4	6	8

^1^ Construction and maintenance of traps included activities such as setting t-posts, wiring panels to posts, hoisting net in air, deploying electronics, changing batteries, and repairing trap parts. ^2^ Baiting was time spent putting bait at each site and travelling between sites. ^3^ Trap observation required for drop nets because this system was user-activated, which required the physical presence of a trapper. ^4^ Removal refers to all activities spent euthanizing and disposing of carcasses. ^5^ Trap observation time was not recorded because a human trapper was not required to be present at the trap site. A real-time alert was sent to the trapper’s phone, allowing the trapper to see what was under the trap. Then, the trapper could send a signal to drop the trap after confirming wild pig presence.

## Data Availability

The data used herein may be made available upon reasonable requests to the Noble Research Institute, LLC by contacting the corresponding author (S.L.W.).
